# Supplementary material for: A Global Screen for Assembly State Changes of the Mitotic Proteome by SEC-SWATH-MS
Source: Cell Syst. 2020 Feb 26;10(2):133–155.e6. doi: 10.1016/j.cels.2020.01.001 (PMC7042714; doi:10.1016/j.cels.2020.01.001)

Q5ZPR3 | CD276_HUMAN | CD276 B7H3 PSEC0249 UNQ309/PRO352
Monomer MW [kDa]: 57.235 Monomer expected elution fraction: 45

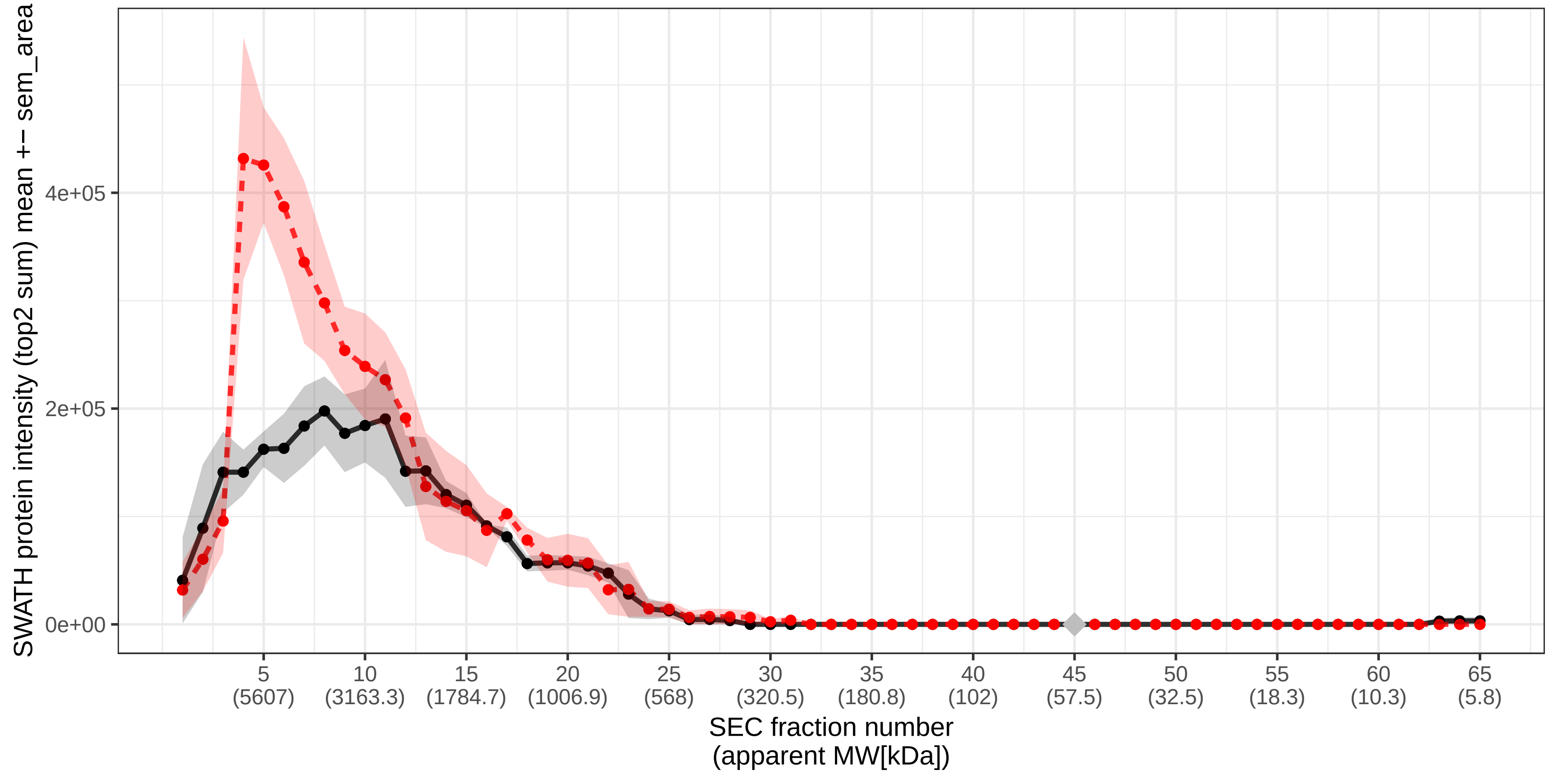

Supplement: Data S1. SEC-SWATH-MS Protein Chromatograms, Related to Figure 1 [file mmc6.zip › SECchrom_Q5ZPR3_CD276_HUMAN_CD276_B7H3_PSEC0249_UNQ309_PRO3.pdf]
